# The Quality of Zero Bounds for Complex Polynomials

**DOI:** 10.1371/journal.pone.0039537

**Published:** 2012-07-12

**Authors:** Matthias Dehmer, Yury Robertovich Tsoy

**Affiliations:** 1 Institute for Bioinformatics and Translational Research, UMIT, Hall in Tyrol, Austria; 2 Department of Computer Engineering, Tomsk Polytechnic University, Tomsk, Russia; Universita’ del Piemonte Orientale, Italy

## Abstract

In this paper, we evaluate the quality of zero bounds on the moduli of univariate complex polynomials. We select classical and recently developed bounds and evaluate their quality by using several sets of complex polynomials. As the quality of priori bounds has not been investigated thoroughly, our results can be useful to find optimal bounds to locate the zeros of complex polynomials.

## Introduction

Deriving zero bounds for real and complex zeros of polynomials is a classical problem that has been proven essential in various disciplines such as engineering, mathematics, and mathematical chemistry [1]–[6]. As indicated, there is a large body of literature dealing with the problem of providing disks in the complex plane representing so-called inclusion radii (bounds) where all zeros of an univariate complex polynomial are situated. Let 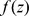 be a an univariate complex polynomial. Then, a crucial question is to investigate how accurate an inclusion radius is, i.e., how well does the bound reflect the real location of the zeros of 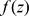 by determining the quantity 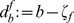 where 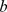 is the bound under consideration and 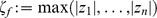. It is clear that the more optimal a zero bound is, the better the value can serve as an estimate to start a numerical procedure such as Newton’s or Sturm’s method [7].

Starting from a set of complex polynomials, it is often difficult to find an optimal bound, i.e., for which 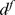 either vanishes or is very little. Another problem is that for many bounds, sharpness results do not exist. Sharpness means there exists a polynomial possessing a zero that lies on the circle which includes all zeros of the polynomial in question. This problem calls for a systematic treatment namely to study the optimality of zero bounds for particular classes of polynomials numerically. To our best knowledge, this problem has not yet been explored properly; see, e.g., [8]. A reason for this is surely the vast amount of existing bounds for locating the zeros of real and complex polynomials [9]–[13]. The only attempt in this direction we got aware of is due McNamee and Olhovsky [8]. They implemented several zero bounds by using 1200 polynomials with random real or complex roots and calculated their values numerically [8]. Among other calculated zero bounds they did not state explicitly in [8], the bounds ue to Deutsch [14] and Kalantari [13] have been evaluated and found to be optimal by using the mentioned set of polynomials [8].

The main contribution of this paper is as follows: In contrast to [8], we evaluate classical and more recently developed zero bounds by using different classes of complex polynomials numerically. Among these classes are also lacunary polynomials and those, whose coefficients satisfy certain conditions by means of inequalities. We calculate several bounds for complex polynomials due to Cauchy [3], Dehmer [1], [9], Kalantari [13], Jain [15], Joyal [10] etc., see Table 1–Table 6. As a result, we find that some of the bounds due to Dehmer, Joyal and Cauchy outperform Kalantari’s bounds by using particular classes of polynomials. This result triggers the hypothesis that it may be worthwhile to further develop bounds for special polynomials (e.g., lacunary polynomials or complex polynomials with special conditions for the polynomials coefficients) which are more optimal than by using general zero bounds. For instance, Theorem (10) developed by Dehmer et al. [9] will prove this hypothesis.

## Methods

In the following, we state the most important zero bounds for locating the zeros of complex polynomials as theorems we are going to use in this study. The quality of these statements will be evaluated in the section ‘Results’. We distinguish two classes of bounds, namely explicit and implicit zero bounds, see [1], [9].

### Explicit Bounds for Complex Polynomials: Classical and Recent Results

The following bounds [4], [16], [17] represent functions of all coefficients of a given polynomial. In fact, this type of zero bound has been called explicit bound [1], [9] as the value of the bound can be calculated explicitly by using quantities based on the moduli of the polynomial coefficients.

**Theorem 1 (Cauchy** [**3**] **)** *Let*

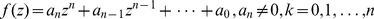



*be a complex polynomial. All zeros of 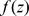 lie in the closed disk 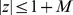, where*

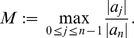



**Theorem 2 (Joyal** [**10**] **)** *Let*

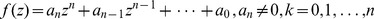



*be a complex polynomial. All zeros 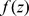 lie in*

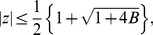
(1)
*where*



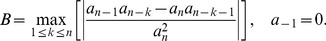
(2)


**Table 1:** Mean bounds values for polynomials 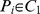.

Bounds	*n* = 5	*n* = 10	*n* = 20	*n* = 30	*n* = 40	*n* = 50	*n* = 60	*n* = 70	*n* = 100
Cauchy, Th. (1)	3.385	4.382	4.515	4.335	4.612	4.729	4.710	4.725	4.812
Cauchy, Th. (13)	2.500	3.006	2.610	2.525	2.577	2.634	2.577	2.614	2.567
Dehmer, Th. (14)	3.367	4.381	4.515	4.335	4.612	4.729	4.710	4.725	4.812
Dehmer, Th. (15)	2.757	3.399	3.084	3.041	3.144	3.218	3.173	3.215	3.205
Dehmer, Th. (16)	3.024	3.902	3.542	3.410	3.568	3.669	3.596	3.665	3.644
Dehmer, Th. (9)	2.788	3.400	3.084	3.041	3.144	3.218	3.173	3.215	3.205
Jain, Th. (5)	19.079	50.691	147.404	286.800	340.555	545.886	744.104	948.732	1481.292
Joyal, Th. (2)	2.648	3.324	3.116	2.998	3.149	3.233	3.176	3.229	3.235
Joyal, Th. (8)	2.684	3.885	4.030	4.148	4.633	4.966	5.066	5.307	5.699
Kalantari, Th. (11)	3.593	4.539	3.755	3.574	3.704	3.793	3.691	3.787	3.679
Kalantari, Th. (12)	2.995	3.777	3.127	3.005	3.082	3.169	3.079	3.160	3.061
Kojima, Th. (4)	7.157	11.544	15.754	22.013	20.301	24.790	26.581	30.713	38.107
Kuniyeda, Th. (6)	3.792	6.441	8.448	9.277	11.177	12.576	13.519	14.291	16.888
Kuniyeda, Th. (7)	3.456	5.101	5.912	6.063	6.909	7.456	7.763	7.995	8.855
Mohammad, Th. (3)	7.871	11.830	16.093	22.262	20.408	24.882	27.215	30.926	38.336

The results are averaged over 1000 independent runs.

**Theorem 3 (Mohammad** [**15**] **)** *Let*

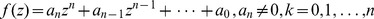



*be a complex polynomial. All zeros 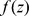 lie in*



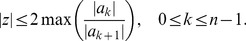
(3)


**Table 2:** Mean bounds values for polynomials 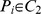.

Bounds	*n* = 5	*n* = 10	*n* = 20	*n* = 30	*n* = 40	*n* = 50	*n* = 60	*n* = 70	*n* = 100
Cauchy, Th. (1)	2.858	2.955	3.128	3.114	3.203	3.224	3.290	3.217	3.364
Cauchy, Th. (13)	2.311	2.331	2.335	2.334	2.374	2.361	2.353	2.327	2.371
Dehmer, Th. (14)	2.837	2.955	3.128	3.114	3.203	3.224	3.290	3.217	3.364
Dehmer, Th. (15)	2.495	2.558	2.610	2.621	2.671	2.671	2.666	2.646	2.702
Dehmer, Th. (16)	2.728	2.806	2.897	2.913	2.999	2.986	3.018	2.968	3.060
Dehmer, Th. (9)	2.526	2.559	2.610	2.621	2.671	2.671	2.666	2.646	2.702
Jain, Th. (5)	14.137	39.253	98.124	192.320	265.311	383.379	579.944	631.977	1059.740
Joyal, Th. (2)	2.390	2.453	2.543	2.559	2.635	2.626	2.661	2.621	2.702
Joyal, Th. (8)	2.421	2.814	3.345	3.652	4.013	4.203	4.431	4.486	5.051
Kalantari, Th. (11)	3.107	3.077	3.080	3.099	3.149	3.138	3.124	3.077	3.158
Kalantari, Th. (12)	2.651	2.632	2.631	2.635	2.688	2.663	2.673	2.625	2.688
Kojima, Th. (4)	5.483	8.052	10.922	14.386	15.662	17.660	22.262	21.553	24.836
Kuniyeda, Th. (6)	3.320	4.506	6.403	7.584	8.964	10.015	11.168	11.606	14.561
Kuniyeda, Th. (7)	3.020	3.564	4.405	4.830	5.386	5.763	6.209	6.279	7.356
Mohammad, Th. (3)	5.898	8.456	11.100	14.728	15.701	17.870	22.372	21.693	24.921

The results are averaged over 1000 independent runs.

**Theorem 4 (Kojima** [**12**] **)** *Let*

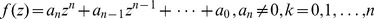



*be a complex polynomial. All zeros 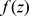 lie in*



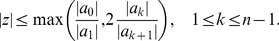
(4)


**Table 3:** Mean bounds values for polynomials 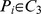.

Bounds	*n* = 5	*n* = 10	*n* = 20	*n* = 30	*n* = 40	*n* = 50	*n* = 60	*n* = 70	*n* = 100
Cauchy, Th. (1)	1.798	1.839	1.862	1.879	1.890	1.893	1.897	1.899	1.903
Cauchy, Th. (13)	1.438	1.484	1.462	1.446	1.433	1.424	1.419	1.410	1.399
Dehmer, Th. (10)	1.513	1.458	1.413	1.393	1.378	1.368	1.363	1.354	1.342
Dehmer, Th. (14)	1.747	1.836	1.862	1.879	1.890	1.893	1.897	1.899	1.903
Dehmer, Th. (15)	1.603	1.689	1.698	1.700	1.703	1.699	1.704	1.698	1.698
Dehmer, Th. (16)	1.642	1.694	1.702	1.703	1.708	1.708	1.710	1.706	1.703
Dehmer, Th. (9)	1.672	1.694	1.698	1.700	1.703	1.699	1.704	1.698	1.698
Jain, Th. (5)	17.221	47.320	135.553	235.369	391.751	549.261	665.022	831.994	1508.291
Joyal, Th. (2)	1.563	1.600	1.607	1.613	1.620	1.621	1.625	1.622	1.623
Joyal, Th. (8)	1.381	1.549	1.726	1.852	1.946	2.030	2.094	2.152	2.290
Kalantari, Th. (11)	1.851	1.937	1.973	1.984	1.989	1.992	1.993	1.995	1.996
Kalantari, Th. (12)	1.562	1.616	1.626	1.627	1.627	1.627	1.627	1.627	1.625
Kojima, Th. (4)	5.941	9.415	14.717	17.694	21.712	25.410	26.355	28.808	35.672
Kuniyeda, Th. (6)	1.613	1.967	2.446	2.837	3.148	3.447	3.678	3.915	4.475
Kuniyeda, Th. (7)	1.819	1.942	2.097	2.224	2.319	2.410	2.476	2.546	2.698
Mohammad, Th. (3)	6.500	9.837	14.935	17.846	21.988	25.515	26.574	28.850	35.818

The results are averaged over 1000 independent runs.

**Theorem 5 (Jain** [**15**] **)** *Let*

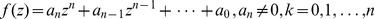



*be a complex polynomial. All zeros 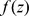 lie in*



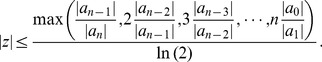
(5)


**Table 4:** Mean bounds values for polynomials 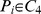.

Bounds	*n* = 5	*n* = 10	*n* = 20	*n* = 30	*n* = 40	*n* = 50	*n* = 60	*n* = 70	*n* = 100
Cauchy, Th. (1)	2.074	2.220	2.439	2.627	2.779	2.896	3.011	3.154	3.395
Cauchy, Th. (13)	1.648	1.730	1.718	1.717	1.699	1.696	1.682	1.664	1.653
Dehmer, Th. (14)	2.038	2.219	2.439	2.627	2.779	2.896	3.011	3.154	3.395
Dehmer, Th. (15)	1.824	1.957	2.047	2.121	2.170	2.209	2.241	2.279	2.354
Dehmer, Th. (16)	1.729	1.871	1.983	2.069	2.123	2.167	2.211	2.249	2.326
Dehmer, Th. (9)	1.874	1.959	2.047	2.121	2.170	2.209	2.241	2.279	2.354
Jain, Th. (5)	11.649	36.042	114.092	221.236	350.677	475.578	665.546	789.016	1323.884
Joyal, Th. (2)	1.668	1.745	1.841	1.932	1.990	2.040	2.085	2.126	2.207
Joyal, Th. (8)	1.456	1.700	2.038	2.321	2.528	2.717	2.888	3.046	3.408
Kalantari, Th. (11)	2.101	2.171	2.183	2.192	2.183	2.176	2.175	2.164	2.152
Kalantari, Th. (12)	1.706	1.767	1.774	1.779	1.776	1.771	1.767	1.761	1.753
Kojima, Th. (4)	4.686	8.516	13.542	17.812	20.859	23.248	26.198	28.486	33.187
Kuniyeda, Th. (6)	1.841	2.444	3.484	4.478	5.371	6.186	7.034	7.872	9.900
Kuniyeda, Th. (7)	1.990	2.262	2.732	3.180	3.571	3.915	4.273	4.629	5.423
Mohammad, Th. (3)	5.042	8.655	13.598	17.865	20.899	23.300	26.280	28.505	33.218

The results are averaged over 1000 independent runs.

**Theorem 6 (Kuniyeda** [**11**] **)** *Let 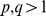 mit* 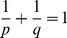. *All zeros of*

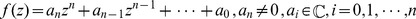

*lie in*

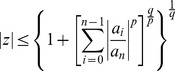
(6)


**Table 5:** Mean bounds values for polynomials 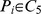.

Bounds	*n* = 5	*n* = 10	*n* = 20	*n* = 30	*n* = 40	*n* = 50	*n* = 60	*n* = 70	*n* = 100
Cauchy, Th. (1)	6.953	8.934	11.459	17.945	18.848	21.994	26.068	23.206	31.766
Cauchy, Th. (13)	3.687	3.682	3.516	3.711	3.782	3.693	3.834	3.616	3.848
Dehmer, Th. (14)	6.945	8.934	11.459	17.945	18.848	21.994	26.068	23.206	31.766
Dehmer, Th. (15)	4.013	4.326	4.598	5.183	5.482	5.662	6.053	5.868	6.637
Dehmer, Th. (16)	4.849	5.353	5.737	7.021	7.440	7.825	8.729	7.984	9.543
Dehmer, Th. (9)	4.028	4.326	4.598	5.183	5.482	5.662	6.053	5.868	6.637
Jain, Th. (5)	13.985	41.487	118.855	217.464	350.344	485.687	626.950	808.537	1557.588
Joyal, Th. (2)	4.230	4.774	5.236	6.535	6.966	7.410	8.294	7.589	9.135
Joyal, Th. (8)	4.568	5.797	7.141	9.749	10.998	12.177	14.254	13.330	17.503
Kalantari, Th. (11)	5.406	5.330	5.016	5.367	5.507	5.286	5.615	5.197	5.664
Kalantari, Th. (12)	4.280	4.221	4.022	4.303	4.386	4.202	4.508	4.151	4.535
Kojima, Th. (4)	7.760	10.959	15.114	18.970	22.702	25.459	25.562	28.069	37.948
Kuniyeda, Th. (6)	8.868	14.608	23.560	43.366	51.404	64.825	82.929	77.652	125.115
Kuniyeda, Th. (7)	7.627	11.174	16.139	27.745	31.332	38.119	47.356	43.228	65.415
Mohammad, Th. (3)	7.942	11.131	15.165	19.162	22.719	25.558	25.616	28.152	37.989

The results are averaged over 1000 independent runs.

**Theorem 7 (Kuniyeda** [**11**] **)** *For 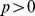, all zeros of*

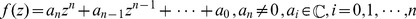



*lie in*

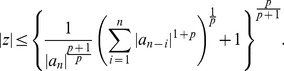
(7)


**Table 6:** Mean bounds values for polynomials 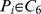.

Bounds	*n* = 5	*n* = 10	*n* = 20	*n* = 30	*n* = 40	*n* = 50	*n* = 60	*n* = 70	*n* = 100
Cauchy, Th. (1)	2.638	2.600	2.637	2.627	2.627	2.607	2.648	2.620	2.634
Cauchy, Th. (13)	1.214	1.093	1.045	1.029	1.022	1.017	1.015	1.012	1.009
Dehmer, Th. (17)	1.216	1.094	1.045	1.030	1.022	1.017	1.015	1.012	1.009
Dehmer, Th. (18)	1.288	1.123	1.059	1.038	1.028	1.022	1.019	1.016	1.011
Dehmer, Th. (19)	2.268	2.262	2.290	2.286	2.278	2.270	2.283	2.246	2.260
Jain, Th. (5)	13.075	22.132	44.164	60.219	92.112	105.597	132.567	173.678	232.253
Joyal, Th. (2)	1.856	1.842	1.856	1.851	1.852	1.845	1.859	1.849	1.853
Joyal, Th. (8)	1.557	1.548	1.559	1.557	1.556	1.549	1.561	1.551	1.555
Kalantari, Th. (11)	2.206	2.087	2.044	2.028	2.021	2.016	2.014	2.012	2.008
Kalantari, Th. (12)	1.785	1.688	1.654	1.641	1.635	1.631	1.630	1.628	1.625
Kojima, Th. (4)	1.813	1.534	1.531	1.391	1.596	1.464	1.531	1.720	1.610
Kuniyeda, Th. (6)	2.166	2.139	2.173	2.169	2.165	2.142	2.178	2.147	2.165
Kuniyeda, Th. (7)	2.363	2.338	2.366	2.363	2.360	2.340	2.372	2.346	2.363
Mohammad, Th. (3)	3.625	3.068	3.061	2.783	3.192	2.928	3.063	3.440	3.220

The results are averaged over 1000 independent runs.

**Theorem 8 (Joyal** [**10**] **)** *For 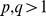 and 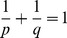, all zeros of*

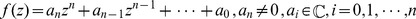

*lie in*

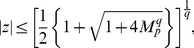
(8)where



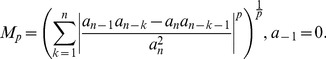
(9)The following explicit bounds have been recently proven by Dehmer and Mowshowitz [9]. It has been shown that they often lead to considerably better values than by using classical ones. Clearly, this depends on the underlying class of polynomials. See also section ‘Results’.

**Theorem 9 (Dehmer** [**9**] **)** *Let*

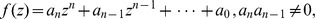

*be a complex polynomial. All zeros of 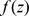 lie in the closed disk*

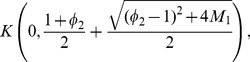
(10)
*where*



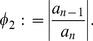
(11)The next theorem gives a bound for polynomials with restrictions on the coefficients. Dehmer [1] has shown that such bounds can be more precise and often lead to better results when locating the zeros of polynomials. See also Table 3.

**Theorem 10 (Dehmer** [**9**] **)** *Let*

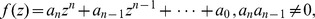

*be a complex polynomial. Suppose that 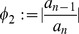 and*



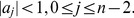
(12)
*All zeros of 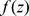 lie in the closed disk*

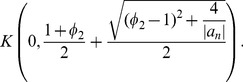
(13)


*The bound is sharp for all polynomials of the form*

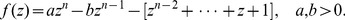
(14)


Further recent results when proving upper bounds have been found by Kalantari [13]. He has found a family of zeros bounds for analytic functions that has been proven powerful when comparing the resulting bounds with classical ones by using complex polynomials [8].

**Theorem 11 (Kalantari** [**13**] **)** *Let 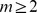 and let 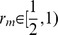 be the positive root of the polynomial*

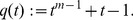
(15)


*For* 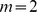 *and* 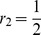, *all zeros of the complex polynomial*

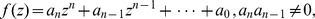

*lie in the closed disk*



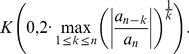
(16)
**Theorem 12 (Kalantari** [**13**] **)** *Let 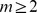 and let 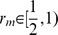 be the positive root of the polynomial*

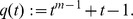



*For* 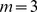 *and* 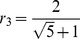, *all zeros of the complex polynomial*

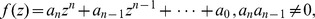

*lie in the closed disk*



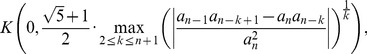
(17)

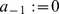
.

### Implicit Bounds for Complex Polynomials: Classical and Recent Results

The bound value of an implicit zero bounds depends on determining the root of a so-called concomitant polynomial [9]. This polynomial can often be obtained from the proof of the underlying theorem. An example thereof is Equation (18).

**Theorem 13 (Cauchy** [**3**] **)** *Let*

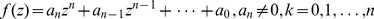

*be a complex polynomial. All zeros of 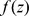 lie in the closed disk 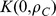, where 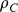 denotes the positive zero of*



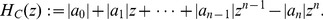
(18)The following implicit zero bounds might be easier to determine (e.g., by hand) when applying this apparatus in practice.

**Theorem 14 (Dehmer** [**1**] **)** *Let*

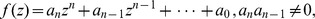

*be a complex polynomial. All zeros of 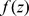 lie in the closed disk 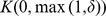 where 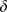 denotes the positive root of the equation*

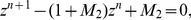
(19)and



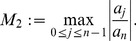
(20)
*The bound is sharp for all polynomials of the form*

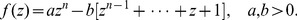
(21)


**Theorem 15 (Dehmer** [**9**] **)** *Let*

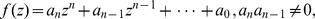

*be a complex polynomial. All zeros of 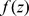 lie in the closed disk 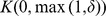 where 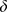 denotes the positive root of the equation*

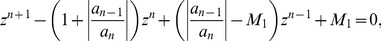
(22)
*and*



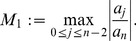
(23)
*The bound is sharp for all polynomials of the form*

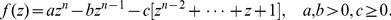
(24)


In particular, the concomitant polynomial of the next theorem is cubical, see Equation (27). This can be beneficial for practical applications as we only have to determine the positive root of a polynomial whose degree equals three.

**Theorem 16 (Dehmer** [**9**] **)** *Let*

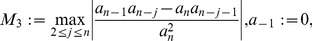
(25)
*and*



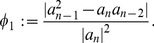
(26)
*In addition, let*

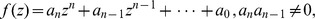

*be a complex polynomial. All zeros of 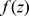 lie in the closed disk 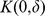 where 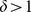 is the largest positive root of the equation*



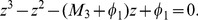
(27)
*Moreover,*

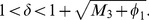
(28)


### Bounds for Special Lacunary Polynomials

In this section, we state bounds [9] for lacunary polynomials, i.e., polynomials in which some coefficients vanish. The hypothesis is that special bounds for lacunary polynomials might lead to better results than by using general zero bounds, see the statements in the previous section.

**Theorem 17 (Dehmer** [**9**] **)** *Le*t
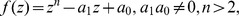

*be a complex polynomial. All zeros of 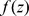 lie in 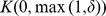, where 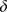 is the unique positive root of the equation*



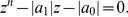
(29)Using the same method of proof we establish the following.

**Theorem 18 (Dehmer** [**9**] **)** *Let*

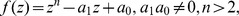

*be a polynomial with arbitrary coefficients. All zeros of 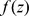 lie in 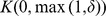, where 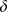 is the unique positive root of the equation*



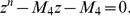
(30)We conclude this section with the following theorem.

**Theorem 19 (Dehmer** [**9**] **)** *Let*

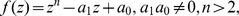

*be a complex polynomial. All zeros of 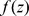 lie in*



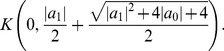
(31)


### Data: Classes of Complex Polynomials

We define the classes of polynomials used in this study as follows (GD stands for Gaussian Distribution).

**Definition 1**

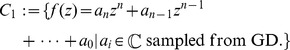
(32)


**Definition 2**

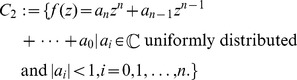
(33)


**Definition 3**

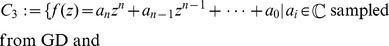





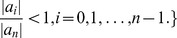
(34)


**Definition 4**

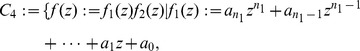


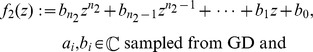


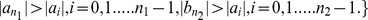
(35)


**Definition 5**

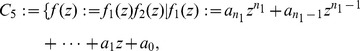


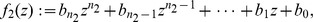


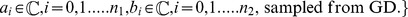
(36)


**Definition 6**

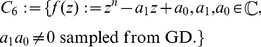
(37)


### Software

We developed a C# program for calculating 19 zero bounds by using complex polynomials. These bounds have been calculated by generating random polynomials based on the following distributions: Gaussian, Poisson, Geometric, and Uniform in [−1, 1]. We used the batch mode of this software to compute all available bounds for a specified number of polynomials having certain degrees. For each class 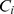 (see section ‘Data: Classes of Complex Polynomials’), we calculate the average by performing 1000 independent runs. To calculate the zeros of the random polynomials, one has to select.

- the degree of a polynomial.
- the type of distribution.
- whether the polynomial is complex or real-valued.

We emphasize that in this study, we only used complex polynomials. After each batch run, the following information is available:

- the type of distribution and distribution parameters.
- the parameter 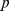 required for some bounds, for example see Equations (6) or (7).
- tables with mean and standard deviation of ranks of the bounds in terms of their optimality for different polynomial orders.
- tables with mean and standard deviation of bound values for different degrees of the polynomials.

Moreover for each polynomial degree, a brief summary of the results for a batch run is available, which includes:

- the type of distribution, distribution parameters and the polynomial degree.
- number of runs.
- the parameter 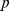, see Equations (6) or (7).
- bound name with the best (worst) sum rank.
- bound name with the worst (largest) sum rank.
- list of the used bounds sorted by their sum rank in ascending order (from best to worst) with information on their sum ranks, minimal and maximal rank achieved during all runs.

Apart from the batch mode, the program can also be used in a single mode. By doing so, the program creates a log file containing the following items:

- the degree of a polynomial.
- the type of distribution.
- the generated complex or real coefficients of the polynomial.
- the parameter 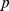, see Equations (6) or (7).
- names of the most sharp and weak bounds with their corresponding values.
- a ranking of the bounds in terms of their optimality in ascending order.

## Results

In this section, we evaluate the quality of the zero bounds presented in the previous sections. We start by observing that Theorem (13) due to Cauchy is often quite sharp. See, for example, the results in Tables 1–4. This is not surprising as this bound is known to be optimal for its class of implicit zero bounds, see [6]. As the numerical results show, this does not mean that other bounds (based on another paradigm) outperform this bound by using special classes of polynomials. This proves our hypothesis that special bounds (e.g., Theorem (10), (19)) may be more suitable and optimal for special classes of polynomials than general bounds (i.e., bounds where no restrictions for the polynomial coefficients are used). In particular, we see that by using the polynomials of Definition (3), the bound due to Dehmer, Theorem (10) outperforms Cauchy’s bound (Theorem (13)) if 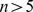. Again, Theorem (10) is based on inequalities involving the polynomial coefficients and leads to a better mean value than by using a general zero bound.

Also, the results by using lacunary polynomials (see Definition (6)) support this hypothesis too. By considering Table 6, we observe that the special bounds for lacunary polynomials due to Dehmer, Theorem (17), (18) perform very similar to Theorem (13) due to Cauchy based on the mean values. In contrast, the explicit bound also developed by Cauchy, Theorem (1) does not give feasible values. Also, the bound due to Jain, Theorem (5) and Kojima, Theorem (4) are not feasible by using the here presented classes of polynomials. This holds for all classes of polynomials used in this study, see Table 1–6. More generally, it has been shown that the inclusion radii given by Theorem (1), (5) are often useless in terms of the real location of the zeros of an underlying polynomial, see [9].

In the following, we discuss some particular cases to find classes of polynomials where the bounds due to Dehmer perform well. Let 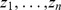 be the zeros of a complex polynomial 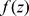 and let 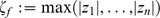. Also, we define the quantity 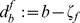, where 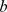 is the corresponding bound value for 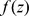. Now, consider the polynomial
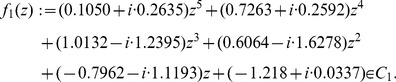
(38)


We yield that Kalantaris bound, Theorem (12) is best, 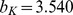 and 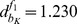. Particularly Table 1 supports the fact that Kalantaris bound, Theorem (12) performs well for 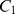 among the used zero bounds. Second best is the bound due to Joyal, Theorem (2), 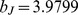 and 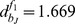. Third best is Dehmer’s bound, Theorem (14), 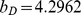 and 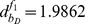. Particularly, Theorem (14) outperforms the classical bound due to Cauchy, Theorem (11), 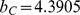 and 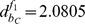. But note that for many other polynomials of 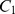, Joyal’s bound, Theorem (2) often was often the best one and Theorem (14) due to Dehmer the second best one. Theorem (14) has the advantage that the positive root of the concomitant polynomial 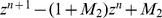 (see Equation (19)) might be easier to determine than by using other bounds which rely on more complex concomitant polynomials, e.g., see Theorem (13).

We already mentioned above that by using special polynomials, some special bounds (based on conditions for the polynomial coefficients) are better suited than by using general zero bounds, e.g., Theorem (1), (2), (11) etc. A positive example for this is the polynomial
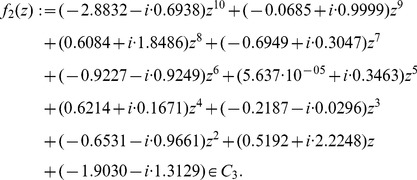
(39)

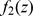
 has the property that its coefficients 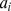 are sampled from a Gaussian Distribution (GD) and it holds 
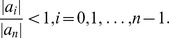
 We yield that the special bound due to Dehmer, Theorem (10) is best 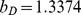 and 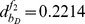. The general zero bounds due to Cauchy and Joyal (Theorem (13) and Theorem (8); for 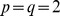) are second best, 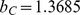, 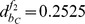 and third best 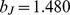, 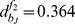. Thus, the special zero bound, Theorem (10) outperforms two classical and general zero bounds by using 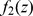. It is clear that the coefficients of the underlying polynomials have a strong impact on the values of the bounds as it be seen by the comparison of 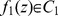 and 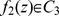. This can be also seen by comparing the quantities 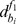 and 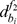. Finally, the chosen bounds are more optimal for 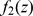 in the sense that the 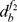 values are much smaller.

Apart from Dehmer’s bounds, we also discuss the quality of the bounds due to Kalantari [13] in a more general context. Note that the Kalantari bounds [8] have already been evaluated and compared with others by McNamee and Olhovsky [8]. In particular, these bounds have been proven efficient for 1200 polynomials with random real or complex roots. We start with general polynomials given by Definition (1). For 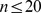, some of Dehmer’s bounds, e.g., Theorem (9), (15) outperform the bound of Kalantari, Theorem (12). If 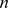 grows, we see that by using other classes the Kalantari bound, Theorem (12) is quite sharp compared to other bounds (except the Cauchy bound, Theorem (13)). The second bound developed by Kalantari, Theorem (11) is worse than almost all Dehmer bounds and others for all 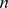. Note that we only evaluated the Kalantari bounds for 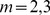; see the underlying concomitant polynomial represented by Equation (15). As a conclusive remark, we find (Table 1–Table 6) that other zero bounds due to Dehmer, Cauchy and Joyal often outperform these bounds. This interesting finding is in contrast to the result due to McNamee and Olhovsky [8], who identified the Kalantari bounds as best when being compared to other classical bounds such as the ones due to Deutsch [14].

### Summary and Conclusion

In this paper, we investigated the quality of zero bounds for complex polynomials numerically. By knowing that the bound values surely depend on the underlying coefficients, we generated several classes of complex polynomials (see section ‘Data: Classes of Complex Polynomials’) to apply the bounds. The set of bounds we have applied consists of (i) classical bounds due to Cauchy [3], Joyal [10], Kuniyeda [11], Kojima [12] etc. and (ii) recently developed bounds due to Dehmer [1], [9] and Kalantari [13]. Note that the just mentioned zero bounds are different to the ones used by McNamee and Olhovsky [8]. Our findings based on the used classes of complex polynomials show that some of the classical results, e.g., Kuniyeda, Kojima and Mohammad are not suitable to locate the zeros optimally. This does not mean that for some other classes or special polynomials, these bounds could perform better. As shown by Rahman and Schmeisser [6], the classical (implicit) zero bound due to Cauchy, Theorem (13) is often optimal within this class of bounds. Thus, it is not surprising that this bound often performs best for our classes. Anyway, we have found other zero bounds which outperformed this bound for particular classes of polynomials. Hence, it would be valuable to derive further special bounds for special classes of polynomials, see also [1], [9].

This study has illustrated some strong and weak points of the used bounds. As conclusion, it seems that there exist only a few zero bounds which give optimal bound values for a variety of complex polynomials. A reason for this is that in view of the vast amount of existing bounds, their quality has only been very little investigated. Also the result where some of the Dehmer bounds (Theorem (17), (18)) outperform the classical (and sharp) Cauchy bound for lacunary polynomials make us conclude that it will be useful to derive further novel bounds for special cases. In fact, special polynomials, i.e., whose coefficients fulfill special conditions often occur in control engineering, algebraic biology and mathematical chemistry.
